# Supplementary material for: Sequence determinants of in cell condensate morphology, dynamics, and oligomerization as measured by number and brightness analysis
Source: Cell Commun Signal. 2021 Jun 5;19:65. doi: 10.1186/s12964-021-00744-9 (PMC8178893; doi:10.1186/s12964-021-00744-9)
Supplement: Supplementary file 2 — Additional file 1. Sequences of wild-type ARF19 and ARF19 QtoS and QtoG variants with the DNA-binding domain, prion-like domain, predicted IDR, and phox/Bemp1 domain annotated. Figure S1. The COILS webserver shows that the QtoG mutant ablates any possible coiled coils. Table S1. Number and Brightness analysis shows that the ARF19 PB1 domain alone does not form higher-order oligomers in protoplasts. [file 12964_2021_744_MOESM2_ESM.docx]

**Supplementary Information:
Sequence determinants of *in cell* condensate assembly morphology, dynamics, and oligomerization as measured by number and brightness analysis**

Ryan. J Emenecker^1,2,3^, Alex S. Holehouse^1,2^*, Lucia C. Strader^2,3,4^*

1. Department of Biochemistry and Molecular Biophysics, Washington University School of Medicine, St. Louis, MO 63110, USA
2. Center for Science and Engineering Living Systems (CSELS), Washington University, St. Louis, MO 63130, USA
3. Center for Engineering Mechanobiology, Washington University, St. Louis, MO 63130, USA
4. Dept. of Biology, Duke University, Durham, NC 27708

* Correspondence: [lucia.strader@duke.edu](mailto:lucia.strader@duke.edu), [alex.holehouse@wustl.edu](mailto:alex.holehouse@wustl.edu)

Sequences of ARF19 and ARF19 variants. For all three variants, the region that was considered the predicted IDR (which is where glutamines were changed to G or S in the QtoG or QtoS variants, respectively) is underlined. The region in ARf19 wildtype that is in bold is the predicted prion-like domain **(PLD)**. The DNA-binding domain as annotated by Uniprot is in blue text. The phox/Bemp1 (PB1) domain as annotated by Uniprot is in orange text.

**>ARF19 wildtype**

MKAPSNGFLPSSNEGEKKPINSQLWHACAGPLVSLPPVGSLVVYFPQGHSEQVAASMQKQ

TDFIPNYPNLPSKLICLLHSVTLHADTETDEVYAQMTLQPVNKYDREALLASDMGLKLNR

QPTEFFCKTLTASDTSTHGGFSVPRRAAEKIFPPLDFSMQPPAQEIVAKDLHDTTWTFRH

IYRGQPKRHLLTTGWSVFVSTKRLFAGDSVLFVRDEKSQLMLGIRRANRQTPTLSSSVIS

SDSMHIGILAAAAHANANSSPFTIFFNPRASPSEFVVPLAKYNKALYAQVSLGMRFRMMF

ETEDCGVRRYMGTVTGISDLDPVRWKGSQWRNLQVGWDESTAGDRPSRVSIWEIEPVITP

FYICPPPFFRPKYPRQPGMPDDELDMENAFKRAMPWMGEDFGMKDAQSSMFPG**LSLVQWM**

**SMQQNNPLSGSATPQLPSALSSFNLPNNFASNDPSKLLNFQSPNLSSANSQFNKPNTVNH**

**ISQQMQAQPAMVKSQQQQQQQQQQHQHQQQQLQQQQQLQMSQQQVQQQGIYNNGTIAVAN**

**QVSCQSPNQPTGFSQSQLQQQSMLPTGAKMTHQNINSMGNKGLSQMTSFAQEMQFQQQLE**

**MHNSSQLLRNQQEQSSLHSLQQNLSQNPQQLQMQQQSSKPSPSQQLQLQLLQKLQQQQQQ**

**QSIPPVSSSLQPQLSALQQTQSHQLQQLLSSQNQQPLAHGNNSFPASTFMQPPQIQVSPQ**

**QQGQMSNKNLVAAGRSHSGHTDGEAPSCSTSPSANNTGHDNVSPTNFLSRNQQQGQ**AASV

SASDSVFERASNPVQELYTKTESRISQGMMNMKSAGEHFRFKSAVTDQIDVSTAGTTYCP

DVVGPVQQQQTFPLPSFGFDGDCQSHHPRNNLAFPGNLEAVTSDPLYSQKDFQNLVPNYG

NTPRDIETELSSAAISSQSFGIPSIPFKPGCSNEVGGINDSGIMNGGGLWPNQTQRMRTY

TKVQKRGSVGRSIDVTRYSGYDELRHDLARMFGIEGQLEDPLTSDWKLVYTDHENDILLV

GDDPWEEFVNCVQNIKILSSVEVQQMSLDGDLAAIPTTNQACSETDSGNAWKVHYEDTSA

AASFNR

**>ARF19 QtoS**

MKAPSNGFLPSSNEGEKKPINSQLWHACAGPLVSLPPVGSLVVYFPQGHSEQVAASMQKQTDFIPNYPNLPSKLICLLHSVTLHADTETDEVYAQMTLQPVNKYDREALLASDMGLKLNRQPTEFFCKTLTASDTSTHGGFSVPRRAAEKIFPPLDFSMQPPAQEIVAKDLHDTTWTFRHIYRGQPKRHLLTTGWSVFVSTKRLFAGDSVLFVRDEKSQLMLGIRRANRQTPTLSSSVISSDSMHIGILAAAAHANANSSPFTIFFNPRASPSEFVVPLAKYNKALYAQVSLGMRFRMMFETEDCGVRRYMGTVTGISDLDPVRWKGSQWRNLQVGWDESTAGDRPSRVSIWEIEPVITPFYICPPPFFRPKYPRQPGMPDDELDMENAFKRAMPWMGEDFGMKDAQSSMFPGLSLVSWMSMSSNNPLSGSATPSLPSALSSFNLPNNFASNDPSKLLNFSSPNLSSANSSFNKPNTVNHISSSMSASPAMVKSSSSSSSSSSSHSHSSSSLSSSSSLSMSSSSVSSSGIYNNGTIAVANSVSCSSPNSPTGFSSSSLSSSSMLPTGAKMTHSNINSMGNKGLSSMTSFASEMSFSSSLEMHNSSSLLRNSSESSSLHSLSSNLSSNPSSLSMSSSSSKPSPSSSLSLSLLSKLSSSSSSSSIPPVSSSLSPSLSALSSTSSHSLSSLLSSSNSSPLAHGNNSFPASTFMSPPSISVSPSSSGSMSNKNLVAAGRSHSGHTDGEAPSCSTSPSANNTGHDNVSPTNFLSRNSSSGSAASVSASDSVFERASNPVSELYTKTESRISSGMMNMKSAGEHFRFKSAVTDSIDVSTAGTTYCPDVVGPVSSSSTFPLPSFGFDGDCSSHHPRNNLAFPGNLEAVTSDPLYSSKDFSNLVPNYGNTPRDIETELSSAAISSQSFGIPSIPFKPGCSNEVGGINDSGIMNGGGLWPNQTQRMRTYTKVQKRGSVGRSIDVTRYSGYDELRHDLARMFGIEGQLEDPLTSDWKLVYTDHENDILLVGDDPWEEFVNCVQNIKILSSVEVQQMSLDGDLAAIPTTNQACSETDSGNAWKVHYEDTSAAASFNR

**>ARF19 QtoG**

MKAPSNGFLPSSNEGEKKPINSQLWHACAGPLVSLPPVGSLVVYFPQGHSEQVAASMQKQTDFIPNYPNLPSKLICLLHSVTLHADTETDEVYAQMTLQPVNKYDREALLASDMGLKLNRQPTEFFCKTLTASDTSTHGGFSVPRRAAEKIFPPLDFSMQPPAQEIVAKDLHDTTWTFRHIYRGQPKRHLLTTGWSVFVSTKRLFAGDSVLFVRDEKSQLMLGIRRANRQTPTLSSSVISSDSMHIGILAAAAHANANSSPFTIFFNPRASPSEFVVPLAKYNKALYAQVSLGMRFRMMFETEDCGVRRYMGTVTGISDLDPVRWKGSQWRNLQVGWDESTAGDRPSRVSIWEIEPVITPFYICPPPFFRPKYPRQPGMPDDELDMENAFKRAMPWMGEDFGMKDAQSSMFPGLSLVGWMSMGGNNPLSGSATPGLPSALSSFNLPNNFASNDPSKLLNFGSPNLSSANSGFNKPNTVNHISGGMGAGPAMVKSGGGGGGGGGGHGHGGGGLGGGGGLGMSGGGVGGGGIYNNGTIAVANGVSCGSPNGPTGFSGSGLGGGSMLPTGAKMTHGNINSMGNKGLSGMTSFAGEMGFGGGLEMHNSSGLLRNGGEGSSLHSLGGNLSGNPGGLGMGGGSSKPSPSGGLGLGLLGKLGGGGGGGSIPPVSSSLGPGLSALGGTGSHGLGGLLSSGNGGPLAHGNNSFPASTFMGPPGIGVSPGGGGGMSNKNLVAAGRSHSGHTDGEAPSCSTSPSANNTGHDNVSPTNFLSRNGGGGGAASVSASDSVFERASNPVGELYTKTESRISGGMMNMKSAGEHFRFKSAVTDGIDVSTAGTTYCPDVVGPVGGGGTFPLPSFGFDGDCGSHHPRNNLAFPGNLEAVTSDPLYSGKDFGNLVPNYGNTPRDIETELSSAAISSQSFGIPSIPFKPGCSNEVGGINDSGIMNGGGLWPNQTQRMRTYTKVQKRGSVGRSIDVTRYSGYDELRHDLARMFGIEGQLEDPLTSDWKLVYTDHENDILLVGDDPWEEFVNCVQNIKILSSVEVQQMSLDGDLAAIPTTNQACSETDSGNAWKVHYEDTSAAASFNR


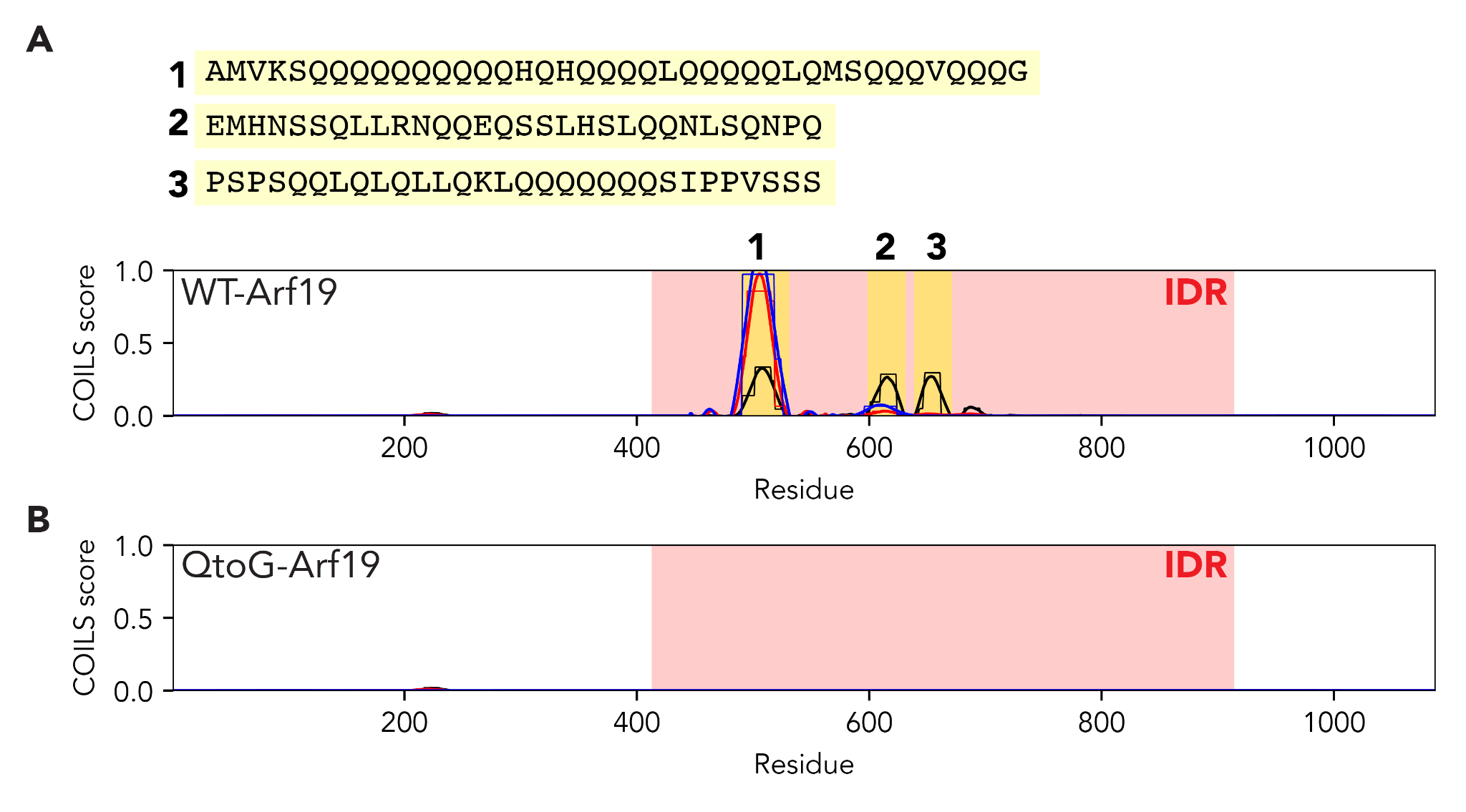


**Figure S1. QtoG mutant abolishes predicted coiled coils.** **(A)** Coiled-coil prediction using the COILS web server (<https://embnet.vital-it.ch/software/COILS_form.html>) identifies three possible regions that may contain predicted coiled-coils, highlighted as regions 1, 2, and 3 with the sequences shown above. Different color lines represent different sized sliding windows (black = 14, red = 21, blue = 28). Coiled-coil prediction analysis was performed using server default values. The Arf19 IDR is highlighted in red. **(B)** Identical analysis performed on the QtoG variant reveals loss of predicted coiled coils across the IDR.

|  | % Monomer | % Dimer | % Trimer | % Tetramer |
| --- | --- | --- | --- | --- |
| Average | 75.403 | 20.842 | 2.959 | 0.796 |
| Standard Deviation | 24.542 | 19.842 | 3.830 | 1.111 |

**Table S1. The ARF19 PB1 domain alone does not form higher order oligomers in protoplasts.** This table contains average values and the standard deviation for various oligomers quantified using Number and Brightness analysis of protoplasts expressing the ARF19 Phox/Bemp1 domain tagged with mVenus. N = 7.
